# Climate-driven morphological change in mosquito vectors: implications for vector competence, distribution and disease transmission—a systematic review

**DOI:** 10.3389/fvets.2026.1956293

**Published:** 2026-10-22

**Authors:** Uzochukwu G. Ekeleme, Ugonma Winnie Dozie, Juliana C. Omire, Ezejindu Cosmas Nnadozie, Onuigbo Martin Chinemerem, Chimezie Christian Iwuala, Nwokeleme Ogechi Chikaodi, Christopher Chike A. Okereke, Uchechukwu M. Chukwuocha

**Affiliations:** 1 Department of Public Health, Federal University of Technology, Owerri, Nigeria; 2 Department of Microbiology, School of Biological Sciences, Federal University of Technology, Owerri, Nigeria; 3 Department of Public Health, Faculty of Allied Health Sciences, David Umahi Federal University of Health Sciences, Uburu, Nigeria; 4 Department of Pharmaceutical Microbiology and Biotechnology, Abia State University, Uturu, Nigeria

**Keywords:** climate change, disease transmission, morphological adaptation, mosquito vectors, phenotypic plasticity, vectorial capacity, wing morphometrics

## Abstract

**Background:**

Climate change is altering the thermal environments experienced by mosquito vectors during development, with consequences for adult morphology and potentially for vector competence and pathogen transmission dynamics.

**Aim:**

This systematic review synthesizes evidence on climate-associated morphological change in mosquito vectors, focusing on body size and wing morphology, and examines how these changes may relate to vectorial capacity, geographic distribution, and disease transmission.

**Methods:**

This systematic review follows the PRISMA 2020 reporting framework and uses the Population, Exposure, Comparator, Outcome (PECO) structure. Searches were conducted in PubMed/MEDLINE (*n* = 31), Elsevier Embase (*n* = 28), Clarivate Web of Science Core Collection (*n* = 22), and CAB Direct/CAB Abstracts (*n* = 14), yielding 95 records. After removal of 34 duplicates, 61 unique records underwent title/abstract screening, 37 underwent full-text assessment, and 24 sources were retained for synthesis.

**Results:**

Twelve primary empirical studies and twelve contextual or methodological sources were included. Across the primary evidence base, warmer developmental conditions generally reduced adult body or wing size, although responses varied by species, population and nutritional environment. The transmission implications were parameter-specific: smaller mosquitoes may exhibit compensatory increases in feeding frequency, whereas larger mosquitoes may show greater competence per infectious bite in some experimental systems. Temperature also affected adult survival and extrinsic incubation in non-linear ways, so no uniform directional effect on transmission could be inferred.

**Conclusion:**

Climate-associated thermal change can alter mosquito morphology through biologically plausible plastic and potentially heritable mechanisms. However, the net effect on pathogen transmission remains contingent on the joint effects of biting frequency, vector competence, survival, development, extrinsic incubation, and local ecological conditions. Wing geometric morphometrics may provide a useful supplementary surveillance measure where validated locally.

**Systematic review registration:**

https://www.crd.york.ac.uk/PROSPERO/view/CRD420261483189, CRD420261483189.

## 1 Introduction

Anthropogenic climate change has ceased to be a distant ecological threat, and instead is actively contributing to modern-day changes in the biology of vectors. In this respect, mosquitoes are at the forefront due to being ectotherms, in which all processes of development and physiology are temperature dependent (1). Not only are rising temperatures, changing precipitation patterns, and increased variability in seasons forcing mosquitoes out of their natural range, but they are also transforming the vectors on an anatomical level – body size, wings, and developmental patterns are undergoing changes. According to Sharma et al. (2), *Aedes aegypti* reared under conditions of warmer temperatures emerge with significantly smaller wing size. Such a pattern can be observed in a broader context, and morphological plasticity in mosquitoes is becoming a part of climate change mechanisms. The current review will explore empirical evidence for the connection between climate-induced warming and the emergence of morphological change in mosquitoes, as well as the controversial issue of vectorial capacity changes induced by those morphological changes.

This review is not solely a matter of entomological interest, given that dengue alone recorded fourteen million documented cases worldwide in 2024, more than twice the number seen the previous year, due partly to the effect of climate on vector dispersal (2). The scientific discourse is split along disciplinary lines: thermal physiologists measure wing size and development time in controlled environmental chambers, epidemiologists estimate transmission risk based on overall climate forecasts, and evolutionary biologists question whether the phenotypic changes they are seeing represent plastic adaptation, genetic assimilation, or developmental stochasticity. This review attempts to tie together these perspectives, as the epidemiological implications of smaller wing size cannot be evaluated until they are understood physiologically, evolutionarily and ecologically.

The knowledge gap that serves as the basis for the present review is characterized by three different levels of evidence, each of which is dealt with independently of the other two: (i) Does warming, which results from climate, lead to morphological change in mosquitoes; (ii) If so, does this morphological change affect physiology or behavior of the mosquitoes; and (iii) If this happens, does this in turn affect vector competency? While evidence regarding one level may be present, this does not automatically prove the subsequent one. Phenotypic plasticity, as defined in this review, refers to the ability of a genotype to have different phenotypes based on the environmental condition during development, which can be reversible without necessitating a change in the allele frequency, while evolutionarily adaptation is the heritable shift in trait values due to selection in different generations. Size reduction in wings and/or body following increased developmental temperatures can be viewed as proof of the plastic response, but in itself, it does not prove evolutionary adaptation, and these are clearly distinguished throughout this review. The main goal of this review, therefore, is to describe the available information on climate-induced responses, both plastic and evolutionary, in mosquitoes; other goals include examining how much these responses are connected with changes in life history characteristics, vectorial capacity, geographic range and ultimately disease transmission.

## 2 Methods

### 2.1 Protocol and registration

This review was prepared following the Preferred Reporting Items for Systematic Reviews and Meta-Analyses (PRISMA 2020) guidance (3). The review protocol was registered in PROSPERO (CRD420261483189) following initial screening and preliminary synthesis. Registration therefore occurred after the commencement of the initial screening and should not be characterized as prospective. The registered protocol subsequently guided the expanded multi-database verification, duplicate screening, data extraction and methodological assessment workflow.

### 2.2 Eligibility criteria: PECO framework

The review question was designed using the PECO format, which is considered as the preferred version of PICO adapted for exposure-based research questions (4). The format of PECO used in the present review is depicted in Table 1.

**Table 1:** PECO framework defining the eligibility criteria for this review.

PECO element	Definition applied in this review
Population/Species (P)	Mosquito species of public health importance as recognized disease vectors, principally Aedes, Anopheles and Culex genera.
Exposure (E)	Elevated or experimentally varied developmental/rearing temperature consistent with climate-change-projected warming trajectories, including associated humidity or precipitation variation where reported.
Comparator (C)	Lower, standard, or ambient/control temperature regimes examined within the same study.
Outcome (O)	Morphological traits (body size, wing length, shape or centroid size), life-history traits (development rate, survival, fecundity, gonotrophic cycle), vector competence, genomic or evolutionary adaptation, and/or climate-linked distribution and disease transmission risk.

Adapted from Morgan et al. (4).

### 2.3 Search strategy

The expanded search was conducted in four bibliographic databases using the nine concept clusters described in the original review. PubMed/MEDLINE was searched through the PubMed interface on 12 July 2026 and returned 31 records; Elsevier Embase was searched on 13 July 2026 and returned 28 records; Clarivate Web of Science Core Collection was searched on 14 July 2026 and returned 22 records; and CAB Direct/CAB Abstracts was searched on 15 July 2026 and returned 14 records. Searches were restricted to English-language records published from 1 January 2000 through the search date and were limited to journal articles or indexed scholarly records; conference abstracts, editorials and book reviews were excluded where the interface permitted. No animal-species or geographic filters were applied. Complete line-by-line strategies are provided in Supplementary Table S1.

### 2.4 Study selection

The four-database search identified 95 records: PubMed/MEDLINE (*n* = 31), Embase (*n* = 28), Web of Science Core Collection (*n* = 22), and CAB Direct/CAB Abstracts (*n* = 14). After 34 duplicate records were removed, 61 unique records remained for title and abstract screening. Twenty-four records were excluded at this stage because they did not directly address mosquito thermal biology, morphology, climate-associated adaptation, or climate-linked distribution/transmission potential. Thirty-seven records therefore underwent full-text assessment. Thirteen full-text records were excluded after eligibility verification: eight were non-peer-reviewed/preprint or conference records and five had authorship or publication provenance that could not be independently verified. Twenty-four sources were retained for synthesis. The full-text exclusion audit is provided in Supplementary Table S2.

### 2.5 Data abstraction

Screening was conducted independently by two reviewers using Rayyan, with disagreements resolved by discussion and, where necessary, adjudication by a third reviewer. Full-text eligibility assessment followed the same duplicate-review process. Data extraction was completed independently by two reviewers using a piloted extraction form, with discrepancies reconciled against the original report. Predefined variables included first author and publication year, country/setting, mosquito species, study design, sample or experimental unit, developmental temperature range, comparator temperature, larval nutritional condition, morphological outcomes, life-history outcomes, vector-competence outcomes, direction and magnitude of effect, statistical significance, and the study’s stated implications for vectorial capacity or transmission.

### 2.6 Outcomes and prioritization

Information about the orientation and uniformity of climate-induced morphological change was analyzed to achieve the first goal of the review: the identification of evidence for climate-induced morphological change in mosquito vectors. Information regarding vector competence, biting habits, extrinsic incubation period, and distribution changes was gathered to achieve the second goal of the review: to determine the extent to which morphological change affects vectorial capacity and risk of human diseases.

### 2.7 Bias assessment

Risk of bias was assessed using the SYRCLE Risk of Bias tool for animal studies, selected because the primary evidence base was dominated by controlled laboratory experiments involving mosquito developmental exposures. Two reviewers independently assessed each primary study after piloting the instrument on three studies. Reviewers were not blinded to authors or journal because these details are integral to assessing experimental methods. Disagreements were resolved by consensus and adjudicated by a third reviewer when consensus could not be reached. The assessment covered sequence generation, allocation and housing, baseline comparability, blinding, incomplete outcome data, selective reporting and other sources of bias. Domains not applicable to a study design were recorded as N/A. The complete study-level matrix is provided in Supplementary Table S3.

### 2.8 Synthesis of results

Results are synthesized qualitatively and thematically because the primary studies differed substantially in mosquito species, thermal exposure, experimental conditions and outcome measurement. Direct empirical evidence is synthesized separately from contextual sources addressing genomic adaptation, distribution, survival modeling, methodological validation and historical climate–disease debates. Effect estimates are presented descriptively where reported; no single pooled meta-analytic estimate is calculated because the outcomes are not sufficiently commensurate.

## 3 Results

### 3.1 Risk-of-bias assessment

Across the 12 primary empirical studies, four were judged to have overall low risk of bias and eight had some concerns; none was classified as having an overall high risk of bias. The most frequent concern was lack of blinded outcome assessment, which is difficult to avoid in laboratory mosquito studies because investigators generally know the exposure assigned to experimental groups. Concerns relating to allocation or housing procedures were more common in older studies. These judgments increase confidence in the direction of the recurring temperature–morphology association but reduce confidence in exact effect magnitudes and extrapolation from laboratory conditions to field populations.

### 3.2 Article searches and screening

The expanded four-database search yielded 95 records: 31 from PubMed/MEDLINE, 28 from Embase, 22 from Web of Science Core Collection, and 14 from CAB Direct/CAB Abstracts. Thirty-four duplicate records were removed, leaving 61 unique records for title/abstract screening. Twenty-four records were excluded at title/abstract screening, leaving 37 full-text records. Thirteen full-text records were excluded after eligibility verification, comprising eight non-peer-reviewed/preprint or conference records and five records for which authorship or publication provenance could not be independently verified. Twenty-four sources were therefore included in the synthesis, of which 12 were classified as primary empirical studies and 12 as contextual or methodological sources.

The numerical pathway reconciles across the review: 31 + 28 + 22 + 14 = 95 records identified; 95–34 duplicates = 61 unique records; 61–24 title/abstract exclusions = 37 full-text records; and 37–13 full-text exclusions = 24 included sources. The 24 included sources were explicitly separated into 12 primary empirical studies and 12 contextual or methodological sources so that contextual literature was not treated as direct evidence for morphological effect estimates (Figure 1).

**Figure 1:**
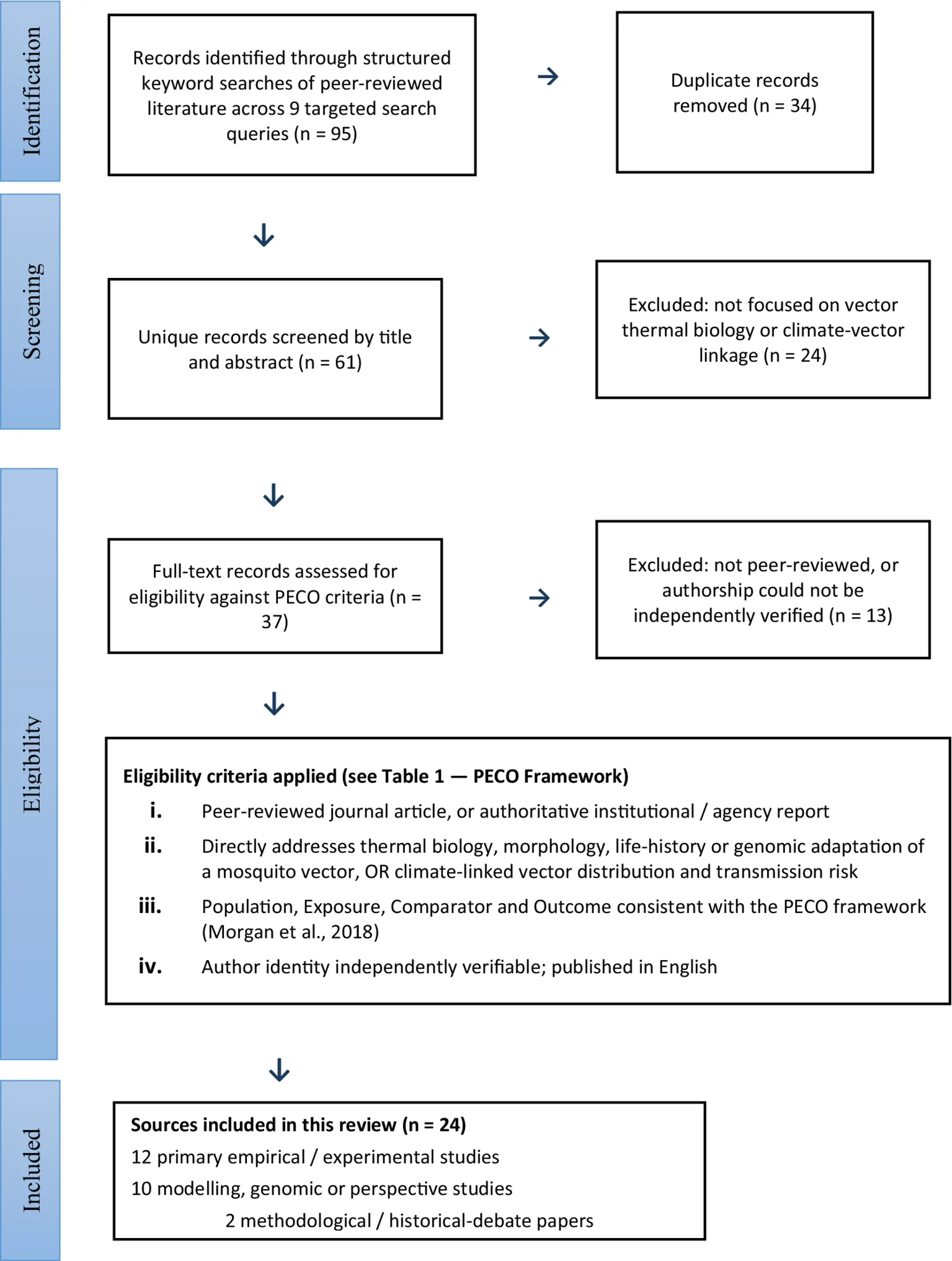
PRISMA flow diagram of study identification and selection. Adapted from the PRISMA 2020 flow diagram template (3).

### 3.3 Overview of the evidence base

Twenty-four sources were included in the synthesis. Twelve were classified as primary empirical studies because they reported direct experimental, observational or field-derived evidence on mosquito morphology, life-history traits, vector competence or thermal adaptation. Twelve additional sources were classified as contextual or methodological because they provided modeling, distributional, mechanistic, taxonomic, methodological or historical evidence without serving as direct primary morphological comparisons. This distinction is maintained throughout the synthesis.

### 3.4 Characteristics of included primary studies

Table 2 summarizes the 12 primary empirical studies underlying the principal evidence synthesis. Contextual sources cited in the narrative are not assigned effect estimates in Table 2. For each primary study, the table reports vector species, evidence type, location/setting, thermal exposure, direction of change, reported magnitude, statistical significance and relevance to vectorial capacity or transmission.

**Table 2:** Characteristics of primary studies.

Citation	Vector species	Evidence type / classification	Location / setting	Temperature / exposure	Direction of change	Magnitude	Statistical significance	Relevance to vectorial capacity/transmission
Sharma et al. (2)	Ae. aegypti	Primary empirical / experimental	Central India; laboratory	20 °C vs. 32 °C; 10–40 °C gradient	Smaller wings and centroid size at 32 °C	Centroid size approximately 18% lower in females and 15% lower in males	*p* < 0.001	Smaller phenotype may increase feeding frequency; survival/fecundity peaked near 26 °C
Chu et al. (5)	Nyssorhynchus darlingi	Primary empirical / experimental	Brazil; four biomes	20, 24 and 28 °C	Body size decreased and development accelerated with warming	Approximately 11–17% smaller at 28 °C than 20 °C, varying by population	*p* < 0.05	Faster development may shorten generation time and alter malaria-vector dynamics
Mohammed and Chadee (25)	Ae. aegypti	Primary empirical / experimental	Trinidad; laboratory	20–34 °C	Development time shortened; survival declined at higher temperatures	Development time approximately 31% shorter at 30 °C than 24 °C	*p* < 0.01	Shorter development can increase turnover, while reduced survival may offset gains
Delatte et al. (26)	Ae. albopictus	Primary empirical / experimental	Réunion Island; laboratory	15–35 °C	Development, survival, fecundity and gonotrophic cycle varied non-linearly	Gonotrophic cycle approximately 28% shorter at 30 °C than 20 °C	*p* < 0.05	Moderate warming may increase feeding frequency; extreme temperatures reduce survival
Grech et al. (27)	Ae. aegypti; Cx. quinquefasciatus	Primary empirical / experimental	Córdoba, Argentina; laboratory	18–30 °C	Species-specific life-history responses	Development time differed by approximately 20–35% across treatments	*p* < 0.05 for selected contrasts	Species-specific responses complicate multi-species outbreak prediction
Yeap et al. (28)	Ae. aegypti	Primary empirical / field-linked experimental	Queensland, Australia; laboratory/field release	Ambient rearing gradient	Wing length and shape reflected adult quality	Approximately 8–12% difference between quality classes	*p* < 0.01	Wing morphometrics can proxy flight performance and survivorship
Alto, Reiskind and Lounibos (29)	Ae. aegypti; Ae. albopictus	Primary empirical / experimental	Florida, USA; laboratory	Standard vs. restricted larval diet	Larger body size associated with altered dengue susceptibility/dissemination	Approximately 1.7-fold higher dissemination odds in larger class	*p* < 0.05	Larger phenotype may increase competence per infectious bite
Huxley et al. (6)	Aedes spp.	Primary empirical / experimental	Laboratory thermal-performance assay	16–34 °C with resource limitation	Temperature response modified by larval resource availability	Thermal-performance estimates differed by approximately 12–20%	Interaction P < 0.05	Nutritional stress modifies morphology and population fitness under warming
Dennington et al. (8)	Ae. aegypti	Primary empirical / evolutionary experiment	Multiple populations; common garden	Contrasting thermal selection regimes	Population-specific thermal performance persisted under common-garden conditions	Approximately 9–14% divergence across selected populations	*p* < 0.01	Supports heritable thermal adaptation in addition to plasticity
Bennett, McMillan and Loaiza (9)	Ae. aegypti	Primary empirical / genomic observational	Panama; field populations	Climate-associated environmental gradients	Genomic differentiation correlated with local climatic conditions	Approximately 6–10% differentiation in key contrasts	FDR-adjusted *p* < 0.05	Supports local adaptation that may modify thermal responses
Phanitchat et al. (7)	Ae. albopictus	Primary empirical / geometric morphometric	Thailand; laboratory and field-derived strains	20, 25 and 30 °C	Laboratory strain declined with warming; field strain peaked at intermediate temperature	Intermediate-temperature wing area approximately 7% greater than high-temperature area	P < 0.05	Population origin alters the morphology–temperature relationship
Couper et al. (10)	Aedes spp.	Primary empirical / evolutionary experiment	Laboratory common-garden populations	Heat-selection gradient	Heritable variation in heat resistance and thermal performance	Adaptive potential approximately 1.2-fold projected warming rate under study assumptions	*p* < 0.01	Evolutionary capacity may modify future morphology and thermal tolerance

### 3.5 Thermal effects on mosquito body size and wing morphology

The influence of the temperature of rearing on adult mosquito size is one of the most reliable findings in the field of medical entomology but it still has quite unexpected interpretations. Using Ae. aegypti specimens collected in central India, Sharma et al. (2) reared the larvae at temperatures ranging from 10 to 40 degrees centigrade and showed that the wings were significantly larger at 20 degrees centigrade than at 32 degrees centigrade, with substantial reductions in centroid size of wings in males and females at the higher temperature. This finding fits into the temperature size rule that was found to hold for ectothermic organisms; warm conditions speed up metabolic processes, so organisms develop faster and stop developing, hence growing, sooner than those under cooler conditions. Smaller body size at higher temperatures of development has also been observed independently in Nyssorhynchus darlingi, the primary vector of malaria in the Amazon basin, in which Chu et al. (5) observed that the mosquitoes raised at 28 degrees Celsius had smaller body sizes compared to those raised at 20 degrees Celsius in four different populations in Brazil, as shown in Table 2 above. It becomes evident from the convergence of the findings between an Aedes mosquito vector for arboviral diseases and an Anopheles group mosquito vector of malaria that size reduction at high temperatures is a general phenomenon among culicid mosquitoes.

However, these studies differ from each other in ways which are analytically interesting as well. Not only did Chu et al. (5) confirm that thermal plasticity of body size differs depending on the biome of origin with low-latitude Amazonian populations reacting differently to warming in terms of development rates compared to high-latitude Mata Atlântica populations, but they showed that the difference in population-level genetic background has an impact on the manifestation of plastic responses. This is an essential nuance since it means that even the most rigorous study conducted on a single population level cannot be used to make general statements about a species as a whole since local adaptation might either enhance or reduce thermal plasticity of the morphological trait under consideration. Similarly, Sharma et al. (2) point out that their results are obtained for a single Bhopal population living in one humid subtropical climatic zone and warn not to generalize their findings regarding the change in wing size to Ae. aegypti populations in other parts of India, in arid and coastal areas. Thermal plasticity differences according to geographic location create a significant source of uncertainty in climate-vector modeling since the models based on one universal thermal performance curve will misrepresent future morphology and population sizes in areas where the population has been locally adapted.

An additional issue is the problem associated with the interaction of temperature and larval nutrition in terms of being difficult to separate. Huxley et al. (6) found that the impact of resource availability for larvae is dependent on temperature in terms of affecting adult morphology and overall population fitness, thus the same temperature condition could lead to different morphologies depending on the availability of nutrients. This is important to note because any controlled experiment done in a laboratory setting such as the one by Sharma et al. (2), is bound to control larval diets to focus on temperature, however, in natural settings temperature and nutritional stress are bound to be dependent on each other. However, the association between temperature and wing morphology is not necessarily monotonic: employing geometric morphometrics on Ae. albopictus at three different temperatures, Phanitchat et al. (7) observed that while a lab-reared strain exhibits the anticipated decline in wing size with rising temperature, a strain collected from the field had the greatest wings at an intermediate temperature, thereby demonstrating that population source may also have a qualitative impact on the temperature-morphology response curve.

### 3.6 Mechanisms underpinning morphological change: plasticity, genetic adaptation and their interaction

Since we have proven the existence of the morphological changes caused by thermal effects, it is vital to explore the underlying causes of this effect, since it would bring about completely different consequences if it is a matter of adaptation or phenotypic plasticity. Phenotypic plasticity is described as the ability of one genotype to produce different phenotypes depending on the surrounding environment and it is considered to be the main cause of wing size reductions described above. Indeed, the authors of this research specifically emphasize that this phenomenon belongs to the field of developmental plasticity, since the mosquitoes used in the research came from one cohort of field-caught insects. Plastic response may take place in one generation, which means an immediate but reversible response to current conditions.

However, a growing number of evolutionary studies challenge any idea that plasticity alone will be enough to protect mosquitoes from persistent heating. The study by Dennington et al. (8) provides evidence of phenotypic adaptation to changes in temperature in the population of Ae. aegypti, and the authors distinguish this kind of adaptation from plasticity by showing heritable difference between populations exposed to different temperatures, which is observed in common-garden setting. In addition, Bennett, McMillan and Loaiza (9) found signatures of local adaptation in the genome of Ae. aegypti that correlated with climate conditions, and hence proved that the role of selection rather than plasticity cannot be overlooked in understanding the evolutionary history of this vector. Together, these two studies substantially strengthen the case that Ae. aegypti possesses genuine adaptive potential, consistent with the species’ relatively short lifespan and large effective population size.

Nevertheless, there should be some caution in viewing adaptive plasticity optimistically because the effectiveness of adaptive plasticity as a way of protecting organisms from environmental variations reduces the necessity for genetic adaptation. There is no information about whether the plasticity shown by Sharma et al. (2) and Chu et al. (5) has been reducing the speed of genetic adaptation found by Dennington et al. (8) and Bennett, McMillan and Loaiza (9), or whether both have been working in harmony. This can only be revealed through selection experiments on successive generations under thermal stress, which have been done more on Drosophila rather than disease-vector mosquitoes. Additional evidence from a sister species, *Aedes sierrensis*, provides a case for the potential ability of genetic adaptation to keep up with the changing environment: Couper et al. (10) found widespread heritable variation for heat resistance and concluded, using a basic evolutionary framework, that the fastest possible rate of adaptation could be faster than the predicted rate of warming, albeit with a number of caveats.

### 3.7 Morphological change, vectorial capacity and implications for disease transmission

The public health significance of climate-associated morphological change depends on how altered size interacts with separate components of vectorial capacity. Smaller mosquitoes may have reduced energy reserves and consequently compensate through more frequent blood-feeding, increasing the number of host contacts per unit time. Conversely, larger mosquitoes have been associated in some experimental systems with greater susceptibility, dissemination or competence per infectious bite. These mechanisms are not contradictory because they operate on different components of transmission: biting frequency influences host contact rate, whereas competence influences the probability of successful pathogen acquisition and transmission per bite.

Temperature additionally affects adult daily survival and extrinsic incubation in non-linear ways. A temperature regime that produces smaller adults may simultaneously alter feeding frequency, survival and pathogen development, while a larger body phenotype under another regime may be associated with different competence and longevity. The net effect on vectorial capacity therefore depends on the joint balance of biting frequency, competence per bite, adult survival and extrinsic incubation rather than on body size alone.

Another complicating factor is the extrinsic incubation period, the time elapsed between a mosquito biting someone and being able to pass on the infection from that bite, which is extremely sensitive to changes in temperature and does not act in an additive manner relative to mosquito survival. The effect of temperature on vector competence is uniquely unimodal, with transmission being maximized in mosquitoes between 28 to 32 degrees Celsius before declining rapidly at more extreme temperatures because extreme heat causes both increased viral reproduction and decreased life span in mosquitoes. The link between the two was established by Mordecai et al. (11), through mechanistic transmission models for dengue, Zika, and chikungunya, showing that there is an optimal transmission within the relatively narrow range of 18 to 34 degrees Celsius, indicating the effects of climate change on the geographical distribution, as opposed to increase in, the risk of disease transmission. Importantly, the thermal optimum is not always equivalent to the optimum for mosquito physical development: according to Sharma et al. (2), the highest levels of survival and fecundity were observed at 26 degrees Celsius, while wing size reduction was evident at 32 degrees Celsius. In light of recent comparisons between mechanistic and machine learning approaches to modeling mosquitoes’ thermal performance, it becomes apparent that mechanistic thermal-performance curves alone may fail to capture all the nuances of temperature dependence in nature (12). In addition to survival and incubation period, temperature influences vector competence by other means, such as by accelerating the age-dependent decay in innate immunity of mosquitoes, which occurs as a consequence of high rearing temperatures (13).

### 3.8 Range shifts, distribution and the redistribution of transmission risk

Morphological change must not be thought of in isolation from the concurrent and far more widely publicized phenomenon of geographic range expansion as a result of climate change since these two processes are causally linked: populations expanding into novel, and often colder, territories are concurrently exposed to a new temperature regime that will, according to the evidence presented above, affect their morphology. Kraemer et al. (14) modeled the historical and future geographic spread of Ae. aegypti and Ae. albopictus and showed that there is already a considerable poleward and altitudinal shift taking place. This conclusion is corroborated by Ryan et al. (15), who modeled the geographic range expansion for both mosquito vectors and predicted considerable expansions into Europe and North America by the middle of the century, although the vectors follow very different paths. More severe warming scenarios lead to an increased exposure of the population to transmission risk by Ae. aegypti mosquitoes but not Ae. albopictus mosquitoes.

There is an analogy in the literature on malaria for which we find similar evidence albeit limited geographically. Paz (16) synthesizes evidence that shows that mosquito vector ranges have moved up in altitude by an average of 6.5 meters each year and poleward by 4.7 kilometers each year from 1898 to 2016, in line with predictions made by climate-linked range-shift theory. This is corroborated by specific highlands-based evidence as discussed in the Discussion section and also by Kaye et al. (17), who used mathematical modeling to show that natural climate fluctuations without long-term secular warming have an impact on the distribution globally of Ae. aegypti.

This synthesis highlights a compound effect of range expansion on morphological change that has not been thoroughly explored in the literature. Whether the morphological plasticity is actively involved in facilitating the range expansion or whether this is a mere consequence of range expansion needs to be tested empirically through a combination of morphometric measurements and invasion front genetic studies.

## 4 Discussion

This review indicates a biologically plausible association between warmer developmental conditions and reductions in adult body and wing size across the principal Aedes and Anopheles evidence base examined. The direction of the morphological response is relatively consistent, but its magnitude is modified by population origin, nutritional conditions and thermal history. Importantly, the evidence does not establish a uniform direction of change in overall transmission risk because morphology, feeding frequency, vector competence, extrinsic incubation and adult daily survival represent distinct temperature-sensitive components of vectorial capacity.

### 4.1 Methodological considerations: laboratory inference, modeling assumptions and the highland malaria controversy

The critical appraisal of this literature will have to take into account the limitations in the methodology of the literature, since the vast majority of the mechanistic knowledge concerning the impact of temperature on mosquito physiology comes from the constant-temperature lab incubations which have very little resemblance to the fluctuating thermal environments in which mosquitoes operate in the wild. This point is admitted openly by Sharma et al. (2), while previous work demonstrates that fluctuations could lead to life history outcomes different from those in the constant-temperature regimes.

This contradiction between lab-based inference and field-scale cause-and-effect attribution becomes evident when considering the issue of highland malaria in East Africa. The initial investigations of Kenya’s highland temperature trends came to contradictory conclusions about the presence of any warming, which was mainly due to data homogenization and selection criteria (18). The issue was later solved using a high-quality long-term data series proving that warming was occurring (19). An additional step in resolving the issue involved a natural experiment in Ethiopia and Colombia that singled out the effect of temperature on malaria incidence (20). The example provides an important lesson in methodology for the literature review on morphological change.

The important lesson provided by the above-mentioned discussion regarding the morphological change research is methodological in nature, and not substantive, and the fact that climate-disease associations that appear to be intuitively convincing and highly appealing are subject to very strict control of possible confounders, verification of temperature data, and, wherever possible, natural experiment or quasi-experimental approach in order to establish any causality. The morphological change studies, reviewed in the current systematic review, have been conducted using controlled lab-based manipulations, which, while possessing high internal validity, possess poor external validity due to the fact that mosquitoes used in the experiments are usually derived from relatively few founders and raised in the absence of ecological complexity such as presence of predators, competition between species, and heterogeneity in larval habitat quality. Paaijmans et al. (21) showed that down-scaling from coarse climate projection data to fine-scale resolution can lead to completely different results of malaria suitability analysis, a lesson which applies to morphological change just as well because regional averages are unlikely to reflect the micro-climate variations relevant for temperature exposure.

### 4.2 Evidence gaps and synthesis

Several evidence gaps remain. First, the primary evidence supports a recurring association between warmer developmental conditions and smaller adult or wing dimensions, but the response is not identical across populations. Second, evidence for phenotypic plasticity and heritable thermal adaptation exists, yet their relative contributions have not been quantified within a common experimental framework. Third, morphology and transmission parameters have rarely been measured together across the same thermal gradient, limiting inference about net vectorial capacity. Fourth, the evidence base remains concentrated in *Aedes aegypti* and a small number of other species, with fewer direct studies of major African malaria vectors and Culex species. Fifth, field validation under fluctuating temperatures, variable larval nutrition and natural ecological conditions remains limited.

Sixth, the geographical and taxonomic extent of morphological studies is limited to being focused excessively on Ae. aegypti and, to a slightly lesser degree, on An. darlingi, whereas comparable information regarding other epidemiologically relevant vectors, like An. gambiae from sub-Saharan Africa and Culex mosquitoes involved in the spread of West Nile virus, is lacking, although those species occur in geographically different habitats, and in the latter case, probably even more climate-sensitive habitats, as highland Anopheles populations. Seventh, the interplay between range expansion and morphological plasticity of populations during invasions, although theoretically interesting, has not been tested in any way yet and is another area in need of longitudinal studies based on population genetics, wing geometric morphometrics and mark-release-recapture studies of mosquito dispersal in the field – methodologies which are already available (22). Taken together, these gaps point to the fact that although the descriptive part concerning climate effects on morphological traits of mosquitoes has been covered rather well already, the epidemiological aspect of this phenomenon is still insufficiently explored: contemporary disease-transmission models usually include temperature only through life history parameters of the vector, such as development and survival rates (23, 24), neglecting morphological traits of the mosquito.

### 4.3 Limitations of this review

The principal limitations of this review concern heterogeneity, external validity and the predominance of controlled laboratory experiments. Many studies use constant temperature regimes that simplify the fluctuating thermal conditions experienced in nature. The number of directly comparable primary morphological studies is also limited, and evidence is concentrated in a small number of mosquito taxa. The expanded search and screening pathway is presented as a fully reconciled working audit in the Supplementary material, while the heterogeneous evidence base still limits generalization of exact effect magnitudes to field populations.

## 5 Conclusion and recommendations for practice

These lessons in methodology from the controversy surrounding the effect of climate on highland malaria further warn against attributing causation to a lack of validated, high-resolution climate data and quasi-experiments. Evidence-based recommendations for professional and public health practice include the following: Vector surveillance programs should adopt wing geometric morphometrics analysis in conjunction with traditional species identification methods (22), to be used as a cheap and early warning tool of local thermal stress. In designing plans for vector control through climate, there is need to take into account the non-linear unimodal relationship between temperature and vector competence, rather than assume that warming causes a linear increase in the risk of transmission. Funders of research should ensure that multi-generational selection studies and combined field/laboratory studies that quantify morphology, vector competence and biting activity are funded. Lastly, in view of the proven geographical differences in thermal plasticity among mosquito populations, national strategies for vector control should invest in empirically characterizing local populations as Sharma et al. (2) did for central India.

## Data Availability

The original contributions presented in the study are included in the article/Supplementary material, further inquiries can be directed to the corresponding author.
