# Supplementary material for: Climate-driven morphological change in mosquito vectors: implications for vector competence, distribution and disease transmission—a systematic review
Source: Front Vet Sci. 2026 Oct 22;13:1956293. doi: 10.3389/fvets.2026.1956293 (PMC13735103; doi:10.3389/fvets.2026.1956293)
Supplement: Supplementary file 1 [file Table_1.docx]

# **Supplementary Materials**

**Supplementary Table S1. Complete database search strategies**

| Database | Platform/interface | Date | Yield | Filters / limits | Line | Verbatim search string |
| --- | --- | --- | --- | --- | --- | --- |
| PubMed | PubMed/MEDLINE | 12 July 2026 | 31 | English; journal articles; 2000–2026 | 1 | ("Climate Change"[Mesh] OR "Global Warming"[Mesh] OR "climate change"[tiab] OR "global warming"[tiab] OR warming[tiab]) |
| PubMed | PubMed/MEDLINE | 12 July 2026 | 31 | English; journal articles; 2000–2026 | 2 | ("Temperature"[Mesh] OR temperature*[tiab] OR thermal[tiab] OR "rearing temperature"[tiab]) |
| PubMed | PubMed/MEDLINE | 12 July 2026 | 31 | English; journal articles; 2000–2026 | 3 | ("Mosquitoes"[Mesh] OR mosquito*[tiab] OR culicid*[tiab] OR Aedes[tiab] OR Anopheles[tiab] OR Culex[tiab]) |
| PubMed | PubMed/MEDLINE | 12 July 2026 | 31 | English; journal articles; 2000–2026 | 4 | (morpholog*[tiab] OR "body size"[tiab] OR "wing size"[tiab] OR "wing length"[tiab] OR morphometric*[tiab]) |
| PubMed | PubMed/MEDLINE | 12 July 2026 | 31 | English; journal articles; 2000–2026 | 5 | ("vector competence"[tiab] OR "vectorial capacity"[tiab] OR "extrinsic incubation period"[tiab] OR EIP[tiab]) |
| PubMed | PubMed/MEDLINE | 12 July 2026 | 31 | English; journal articles; 2000–2026 | 6 | ("range expansion"[tiab] OR "range shift"[tiab] OR distribution[tiab] OR "distribution shift"[tiab]) |
| PubMed | PubMed/MEDLINE | 12 July 2026 | 31 | English; journal articles; 2000–2026 | 7 | ("highland malaria"[tiab] OR (altitudinal[tiab] AND malaria[tiab])) |
| PubMed | PubMed/MEDLINE | 12 July 2026 | 31 | English; journal articles; 2000–2026 | 8 | 1 AND 2 AND 3 AND 4 |
| PubMed | PubMed/MEDLINE | 12 July 2026 | 31 | English; journal articles; 2000–2026 | 9 | 8 AND (5 OR 6 OR 7) |
| Embase | Elsevier Embase | 13 July 2026 | 28 | English; journal articles; 2000–2026 | 1 | 'climate change'/exp OR 'global warming'/exp OR 'climate change':ti,ab OR 'global warming':ti,ab OR warming:ti,ab |
| Embase | Elsevier Embase | 13 July 2026 | 28 | English; journal articles; 2000–2026 | 2 | 'temperature'/exp OR temperature*:ti,ab OR thermal:ti,ab OR 'rearing temperature':ti,ab |
| Embase | Elsevier Embase | 13 July 2026 | 28 | English; journal articles; 2000–2026 | 3 | 'mosquito'/exp OR mosquito*:ti,ab OR culicid*:ti,ab OR aedes:ti,ab OR anopheles:ti,ab OR culex:ti,ab |
| Embase | Elsevier Embase | 13 July 2026 | 28 | English; journal articles; 2000–2026 | 4 | morpholog*:ti,ab OR 'body size':ti,ab OR 'wing size':ti,ab OR 'wing length':ti,ab OR morphometric*:ti,ab |
| Embase | Elsevier Embase | 13 July 2026 | 28 | English; journal articles; 2000–2026 | 5 | 'vector competence':ti,ab OR 'vectorial capacity':ti,ab OR 'extrinsic incubation period':ti,ab OR eip:ti,ab |
| Embase | Elsevier Embase | 13 July 2026 | 28 | English; journal articles; 2000–2026 | 6 | 'range expansion':ti,ab OR 'range shift':ti,ab OR distribution:ti,ab OR 'distribution shift':ti,ab |
| Embase | Elsevier Embase | 13 July 2026 | 28 | English; journal articles; 2000–2026 | 7 | 'highland malaria':ti,ab OR (altitudinal:ti,ab AND malaria:ti,ab) |
| Embase | Elsevier Embase | 13 July 2026 | 28 | English; journal articles; 2000–2026 | 8 | 1 AND 2 AND 3 AND 4 |
| Embase | Elsevier Embase | 13 July 2026 | 28 | English; journal articles; 2000–2026 | 9 | 8 AND (5 OR 6 OR 7) AND [english]/lim AND [2000-2026]/py |
| Web of Science | Clarivate Web of Science Core Collection | 14 July 2026 | 22 | English; articles; 2000–2026 | 1 | TS=("climate change" OR "global warming" OR "warming trend*") |
| Web of Science | Clarivate Web of Science Core Collection | 14 July 2026 | 22 | English; articles; 2000–2026 | 2 | TS=(temperature OR thermal OR "rearing temperature") |
| Web of Science | Clarivate Web of Science Core Collection | 14 July 2026 | 22 | English; articles; 2000–2026 | 3 | TS=(mosquito* OR culicid* OR Aedes OR Anopheles OR Culex) |
| Web of Science | Clarivate Web of Science Core Collection | 14 July 2026 | 22 | English; articles; 2000–2026 | 4 | TS=(morpholog* OR "body size" OR "wing size" OR "wing length" OR morphometric*) |
| Web of Science | Clarivate Web of Science Core Collection | 14 July 2026 | 22 | English; articles; 2000–2026 | 5 | TS=("vector competence" OR "vectorial capacity" OR "extrinsic incubation period" OR EIP) |
| Web of Science | Clarivate Web of Science Core Collection | 14 July 2026 | 22 | English; articles; 2000–2026 | 6 | TS=("range expansion" OR "range shift" OR distribution OR "distribution shift") |
| Web of Science | Clarivate Web of Science Core Collection | 14 July 2026 | 22 | English; articles; 2000–2026 | 7 | TS=("highland malaria" OR (altitudinal AND malaria)) |
| Web of Science | Clarivate Web of Science Core Collection | 14 July 2026 | 22 | English; articles; 2000–2026 | 8 | 1 AND 2 AND 3 AND 4 |
| Web of Science | Clarivate Web of Science Core Collection | 14 July 2026 | 22 | English; articles; 2000–2026 | 9 | 8 AND (5 OR 6 OR 7) |
| CAB Direct | CAB Direct / CAB Abstracts | 15 July 2026 | 14 | English; journal articles; 2000–2026 | 1 | ("climate change" OR "global warming" OR "warming trend*") |
| CAB Direct | CAB Direct / CAB Abstracts | 15 July 2026 | 14 | English; journal articles; 2000–2026 | 2 | (temperature OR thermal OR "rearing temperature") |
| CAB Direct | CAB Direct / CAB Abstracts | 15 July 2026 | 14 | English; journal articles; 2000–2026 | 3 | (mosquito* OR culicid* OR Aedes OR Anopheles OR Culex) |
| CAB Direct | CAB Direct / CAB Abstracts | 15 July 2026 | 14 | English; journal articles; 2000–2026 | 4 | (morpholog* OR "body size" OR "wing size" OR "wing length" OR morphometric*) |
| CAB Direct | CAB Direct / CAB Abstracts | 15 July 2026 | 14 | English; journal articles; 2000–2026 | 5 | ("vector competence" OR "vectorial capacity" OR "extrinsic incubation period" OR EIP) |
| CAB Direct | CAB Direct / CAB Abstracts | 15 July 2026 | 14 | English; journal articles; 2000–2026 | 6 | ("range expansion" OR "range shift" OR distribution OR "distribution shift") |
| CAB Direct | CAB Direct / CAB Abstracts | 15 July 2026 | 14 | English; journal articles; 2000–2026 | 7 | ("highland malaria" OR "altitudinal malaria") |
| CAB Direct | CAB Direct / CAB Abstracts | 15 July 2026 | 14 | English; journal articles; 2000–2026 | 8 | 1 AND 2 AND 3 AND 4 |
| CAB Direct | CAB Direct / CAB Abstracts | 15 July 2026 | 14 | English; journal articles; 2000–2026 | 9 | 8 AND (5 OR 6 OR 7) |

**Supplementary Table S2. Full-text exclusion audit**

| ID | Citation-style record | Title | Explicit exclusion reason |
| --- | --- | --- | --- |
| FT01 | Adeyemi et al., 2020 | Thermal plasticity and developmental responses of Aedes vectors | Non-peer-reviewed preprint |
| FT02 | Khan et al., 2021 | Climate variability and mosquito abundance in peri-urban environments | Conference abstract; no peer-reviewed journal article located |
| FT03 | Moyo et al., 2019 | Temperature effects on mosquito flight activity | Technical report; not a peer-reviewed journal publication |
| FT04 | Rahman et al., 2022 | Warming and vector competence in tropical mosquitoes | Preprint; peer-reviewed status not established |
| FT05 | Okafor et al., 2018 | Seasonal climate and mosquito density in West Africa | Institutional report; publication provenance not independently verified |
| FT06 | Silva et al., 2020 | Morphometric variation among tropical mosquito populations | Authorship/publication provenance could not be independently verified |
| FT07 | Mensah et al., 2023 | Thermal ecology of disease vectors under urban warming | Non-peer-reviewed repository manuscript |
| FT08 | Patel et al., 2017 | Climate-driven changes in vector behaviour | Conference proceedings without verifiable peer-reviewed journal version |
| FT09 | Garcia et al., 2021 | Mosquito thermal tolerance under projected climate change | Preprint; peer-reviewed publication could not be verified |
| FT10 | Ndlovu et al., 2020 | Wing morphology of mosquitoes in changing environments | Authorship and journal provenance could not be independently verified |
| FT11 | Ibrahim et al., 2019 | Global warming and vector-borne disease emergence | Non-peer-reviewed policy report |
| FT12 | Thomas et al., 2022 | Temperature-mediated changes in mosquito development | Authorship/publication provenance could not be independently verified |
| FT13 | Wilson et al., 2021 | Climate-sensitive mosquito traits: experimental observations | Non-peer-reviewed conference paper |

**Supplementary Table S3. Study-level risk-of-bias assessment**

| Study | Domain-level SYRCLE judgements | Overall RoB | Interpretive note |
| --- | --- | --- | --- |
| Sharma et al., 2025 | Sequence generation: Low; Allocation/housing: Some concerns; Blinding: High; Incomplete outcome data: Low; Selective reporting: Low; Other bias: Some concerns; Applicability: Some concerns | Some concerns | Overall judgement based on the pattern of domain-level concerns and study design. |
| Chu et al., 2019 | Sequence generation: Low; Allocation/housing: Low; Blinding: High; Incomplete outcome data: Low; Selective reporting: Low; Other bias: Some concerns; Applicability: Low | Some concerns | Overall judgement based on the pattern of domain-level concerns and study design. |
| Mohammed and Chadee, 2011 | Sequence generation: Some concerns; Allocation/housing: Some concerns; Blinding: High; Incomplete outcome data: Low; Selective reporting: Low; Other bias: Low; Applicability: Some concerns | Some concerns | Overall judgement based on the pattern of domain-level concerns and study design. |
| Delatte et al., 2009 | Sequence generation: Low; Allocation/housing: Some concerns; Blinding: High; Incomplete outcome data: Low; Selective reporting: Low; Other bias: Low; Applicability: Some concerns | Some concerns | Overall judgement based on the pattern of domain-level concerns and study design. |
| Grech et al., 2015 | Sequence generation: Low; Allocation/housing: Low; Blinding: High; Incomplete outcome data: Low; Selective reporting: Low; Other bias: Low; Applicability: Some concerns | Some concerns | Overall judgement based on the pattern of domain-level concerns and study design. |
| Yeap et al., 2013 | Sequence generation: Some concerns; Allocation/housing: Some concerns; Blinding: High; Incomplete outcome data: Low; Selective reporting: Some concerns; Other bias: Low; Applicability: Some concerns | Some concerns | Overall judgement based on the pattern of domain-level concerns and study design. |
| Alto, Reiskind and Lounibos, 2008 | Sequence generation: Low; Allocation/housing: Low; Blinding: High; Incomplete outcome data: Low; Selective reporting: Low; Other bias: Low; Applicability: Low | Low | Overall judgement based on the pattern of domain-level concerns and study design. |
| Huxley et al., 2021 | Sequence generation: Low; Allocation/housing: Low; Blinding: High; Incomplete outcome data: Low; Selective reporting: Low; Other bias: Low; Applicability: Some concerns | Some concerns | Overall judgement based on the pattern of domain-level concerns and study design. |
| Dennington et al., 2024 | Sequence generation: Low; Allocation/housing: Low; Blinding: High; Incomplete outcome data: Low; Selective reporting: Low; Other bias: Some concerns; Applicability: Low | Some concerns | Overall judgement based on the pattern of domain-level concerns and study design. |
| Bennett, McMillan and Loaiza, 2021 | Sequence generation: Low; Allocation/housing: N/A; Blinding: N/A; Incomplete outcome data: Low; Selective reporting: Some concerns; Other bias: Low; Applicability: Some concerns | Some concerns | Overall judgement based on the pattern of domain-level concerns and study design. |
| Phanitchat et al., 2019 | Sequence generation: Low; Allocation/housing: Some concerns; Blinding: High; Incomplete outcome data: Low; Selective reporting: Low; Other bias: Low; Applicability: Some concerns | Some concerns | Overall judgement based on the pattern of domain-level concerns and study design. |
| Couper et al., 2025 | Sequence generation: Low; Allocation/housing: Low; Blinding: High; Incomplete outcome data: Low; Selective reporting: Low; Other bias: Low; Applicability: Low | Low | Overall judgement based on the pattern of domain-level concerns and study design. |

**Supplementary File S1. PRISMA 2020 checklist — manuscript location map**

| PRISMA item | Requirement | Manuscript location | Status |
| --- | --- | --- | --- |
| 1 Title | Identify report as systematic review | Title, p. 1 | Reported |
| 2 Abstract | Structured summary | Abstract, pp. 1–2 | Reported |
| 3 Rationale | Rationale for review | Introduction, pp. 3–4 | Reported |
| 4 Objectives | Explicit objectives | Introduction, p. 4 | Reported |
| 5 Eligibility | Eligibility criteria | Methods—PECO, p. 4 | Reported |
| 6 Information sources | Databases and dates | Methods—Search strategy, p. 5; S1 | Reported |
| 7 Search strategy | Full search strategies | Supplementary Table S1, p. 27 | Reported |
| 8 Selection process | Screening process | Methods—Study selection, p. 5 | Reported |
| 9 Data collection | Extraction process | Methods—Data abstraction, p. 6 | Reported |
| 10 Data items | Variables extracted | Methods—Data abstraction, p. 6 | Reported |
| 11 Risk of bias | Tool and process | Methods—Bias assessment, p. 6; S3 (p. 32) | Reported |
| 12 Effect measures | Effect measures where applicable | Methods—Synthesis, p. 7 | Reported |
| 13 Synthesis | Synthesis approach | Methods—Synthesis, p. 7 | Reported |
| 14 Reporting bias | Reporting-bias consideration | Discussion, p. 19 | Reported |
| 15 Certainty | Certainty approach where applicable | Discussion, p. 19 | Not applicable |
| 16 Selection results | Numbers at each stage | Results, pp. 7–8; Fig. 1 | Reported |
| 17 Characteristics | Included-study characteristics | Table 2, pp. 10–12 | Reported |
| 18 RoB results | Study-level judgements | Results, p. 7; S3 (p. 32) | Reported |
| 19 Individual results | Individual study findings | Results, pp. 13–16 | Reported |
| 20 Synthesis results | Results of syntheses | Results/Discussion, pp. 13–19 | Reported |
| 21 Reporting biases | Evidence-base limitations | Discussion, p. 19 | Reported |
| 22 Certainty | Certainty of evidence | Discussion, p. 19 | Not applicable |
| 23 Discussion | Interpretation and limitations | Discussion, pp. 17–19 | Reported |
| 24 Registration | Registration and chronology | Methods, p. 4 | Reported |
| 25 Support | Funding/support | Declarations, p. 20 | Reported |
| 26 Competing interests | Competing interests | Declarations, p. 20 | Reported |
| 27 Availability | Data/material availability | Declarations, p. 20 | Reported |
